# The Behavioral and Neurochemical Changes Induced by Boldenone and/or Tramadol in Adult Male Rats

**DOI:** 10.1007/s11064-022-03827-2

**Published:** 2022-11-30

**Authors:** Noha A. Mowaad, Marwa E. A. El-Shamarka, Yasser A. Khadrawy

**Affiliations:** 1 Department of Narcotics, Ergogenic Aids and Poisons,Medical Research and Clinical Studies Institute, National Research Centre, Giza, Egypt; 2 Medical Physiology Department, Medical Research and Clinical Studies Institute, National Research Centre, El-Behouth St, Giza, Egypt

**Keywords:** Boldenone, Tramadol, Cortex, oxidative stress, Neuroinflammation, rat

## Abstract

Boldenone and tramadol are abused among large sectors of adolescents. Therefore, the behavioral changes concerned with memory and cognitive functions and neurochemical variations were investigated in the cortex of rats treated with boldenone and/or tramadol. Rats were divided into control and rats treated with boldenone, tramadol, or both drugs. At the end of the treatment period, the memory and cognitive functions were evaluated by the Y-maze test (YMT) and elevated plus maze test (EPMT) and the motor activity was determined by the open field test (OFT). The cortex was dissected to carry out the neurochemical analyses. Rats treated with boldenone and/or tramadol showed impaired memory and cognitive functions and reduced motor activity. A significant increase in lipid peroxidation (MDA), nitric oxide (NO), and a significant decrease in reduced glutathione (GSH) were observed in the cortex of rats treated with boldenone and/or tramadol. The levels of acetylcholinesterase (AChE) and monoamine oxidase (MAO) decreased significantly. Western blot data showed a significant decrease in Bcl2 and a significant increase in caspase-3 and inducible nitric oxide synthase (iNOS) in rats treated with boldenone and/or tramadol. These changes were associated with neuronal death as indicated from the histopathological examination.

The present findings indicate that boldenone and/or tramadol induced impairment in memory and cognitive functions. These changes could be mediated by the increase in oxidative stress, neuroinflammation, reduced AChE level, and reduced number of survived neurons in the cortex as indicated from the decreased Bcl2 level and the histological examination.

## Introduction

Today, substance abuse represents a major problem afflicting societies and governments. Numerous studies raised concerns about the severe physical and mental damage resulting from drug abuse [1, 2]. Testosterone-derived molecules as anabolic–androgenic steroids (AAS) are often abused by athletes and adolescents who wish to obtain quick muscle masses to improve their performance and for esthetic purposes. The consumption of AAS has become pandemic all over the world [3].

Boldenone (1,4-androstadiene-17b-ol-3-one) is an androgenic steroid used by athletes and body builders to develop a better physical performance and to enhance muscle mass.

It acts by increasing positive nitrogen balance through enhancing protein anabolism and decreasing protein catabolism besides the retention of water, nitrogen, and electrolytes [4].

Despite being banned in humans, boldenone is still available illegally and heavily used by athletes and body builders and for fitness purposes in non-athletics [5]. Several health disorders have been associated with boldenone misuse like renal damage [6], cardiovascular disorders [7], liver dysfunction [8], and testicular problems [9] These actions were attributed to the adverse effects of boldenone on the antioxidant mechanisms of these organs [7]. In addition, a wide range of behavioral and neurochemical alterations have been reported in the nervous system after AAS consumption [10]. It has been reported that 23% of AAS athlete abusers met the diagnostic criteria for major depressive and bipolar disorders especially after regular intake and higher doses, compared to naïve athletes who showed 4% prevalence only for major mood disorders [11]. Moreover, 16% of male AAS abusers had a history of anxiety disorders, panic disorder, obsessive–compulsive disorder, post-traumatic stress disorder, or social phobia [12]. Anxiety and aggressiveness have also been reported by Bueno et al. among AAS users [13]. These effects were mediated by changing neurotransmitter levels in the CNS [14, 15]. Androgen receptors are widely expressed in different brain structures including the hippocampus, amygdala, and cerebral cortex. It has been demonstrated that the use of AAS interferes with important signaling neurotransmitter systems, such as the glutamatergic [16], cholinergic [17], and opioid systems that are known to modulate animal behavior [18].

In addition, AAS could induce excitotoxic neuronal death by interacting with N-methyl-D-aspartate (NMDA) [19, 20] and modulating the redox status in the cortex and hippocampus [13].

Another substance that is highly abused is tramadol. In the last few years, tramadol abuse has steadily increased in Egypt and Middle East countries [21]. Tramadol prevalence in these countries may be due to its cheapness, wide availability and illegal smuggling [22]. Tramadol is used as an effective oral therapy to relief pain in painful diabetic neuropathy [23]. It is an opioid codeine analogue [24]. However, long-term abuse can induce several neurological effects as nervousness, insomnia, anxiety, euphoria, apathy, sleep disorders, agitation, depression, emotional instability, and rarely nightmares, dependency, hallucinations, withdrawal syndrome, suicidal propensity and alteration in cognition function [25–27]. Signs and symptoms of serotonin syndrome, seizures, elevated intracranial pressure, respiratory depression, hepatic diseases as cirrhosis, renal dysfunction, gastrointestinal disorders, cardiovascular diseases, and nervous non-mental disorders have also been reported with tramadol use [28, 29]. It has been concluded that neurotoxicity and neurodegeneration underlie several symptoms induced by tramadol [30]. Although rats treated with tramadol exhibited lower levels of anxiety, they suffered from severe motor impairment which may be due to prefrontal cortical dysfunction [31]. Oxidative stress and inflammation together with marked dose-dependent up-regulation of gene and protein expression of the pro-apoptotic markers Bax and p53 were found in the cerebrum of tramadol-treated rats, accompanied by a decline in the gene and protein expression of the anti-apoptotic marker Bcl-2. This demonstrates the ability of tramadol to trigger apoptotic cell death in rats [32]. Athletes consuming boldenone to accelerate their muscle-building may have to use tramadol to overcome the pain that arises as a result of severe exercises. This may expose them to the hazardous effects induced by the co-administration of boldenone and tramadol.

Due to the serious side effects induced by the wide abuse of boldenone and/or tramadol, the present study was designed to investigate the impact of these two agents on cognitive functions and memory and on the neurochemical changes that may underlie these behavioral effects.

## Material and Methods

### Experimental Animals

Adult male Wistar albino rats, weighing 250–350 g, were used as experimental animals in the present investigation. Rats were obtained from the animal house of the National Research Centre, Egypt. Animals were kept under normal light/dark cycle (12/12) and at controlled temperature (25 ± 2 °C). Standard food pellets and tap water were available for animals during the course of the study ad libitum. All experimental procedures followed the international standard animal ethics applied by the local committee of animal care and use of the National Research Centre (NIH Publications No. 8023, revised 1985).

### Chemicals and Reagents

Bodenone decontate was purchased from Equi-gan® (Lab Tornel, Co., Mexico) and tramadol was obtained from Ministry of Justice, Egypt. Ethanol (90%) (El-Nasr Pharmaceutical chemical company, Egypt), thiobarbituric acid and trichloroacetic acid (Sigma-Aldrich), perchloric acid (Sd fine-chem limited, India),5,5′-Dithiobis(2-nitrobenzoic acid) (Sigma-Aldrich), naphthyl)ethylenediamine dihydrochloride (Sigma), and sulfanilamide (Sigma) were used in the preparation of reagents.

### Experimental Design

In the present study, Wistar adult male rats (28 rats) were divided randomly into four groups (n = 7 in each group). The first group is the control that received isotonic saline solution (sodium chloride solution 0.9%) daily for 2 months. The second group received an intramuscular injection of boldenone decanoate (5 mg/kg) once weekly for 2 months. The third group received a daily intraperitoneal injection of tramadol (20 mg/kg) for 2 months. The fourth group received both boldenone (5 mg/kg/week) and tramadol (20 mg/kg/day) for 2 months. At the end of experiment, rats were subjected to the behavioral tests. The rats were then decapitated and the cerebral cortex of each rat was dissected out and used to carry out the neurochemical and histological analyses.

The dose of boldenone was selected according to the study of Bueno et al. [13]. The present tramadol dose was based on the study of Nagakannan et al., [33]. These studies investigated the behavioral and neurochemical changes induced by these agents.

### Samples Collection

After decapitation, the brain of each rat was removed on ice cold Petri dish. Then the cortex was dissected out, weighed and stored at−80 °C until carrying out the biochemical analyses.

### Behavioral Tests

#### Y-Maze Test (YMT)

This test evaluates the short-term memory [34]. The apparatus employed in the present investigation consisted of a metallic Y-maze comprising three arms, forming the Y shape. Each arm was 35 cm long, 25 cm high, and 10 cm wide and was positioned at 120° extending from a central platform. Normal rodents typically prefer to investigate a new arm of the maze rather than the familiar one. The test was carried out on two successive days. On the first day, which was designated for training, each rat was placed at the central platform and allowed to move freely through the maze for 8 min. On the test day, the sequence of arms entered by each mouse was recorded during the 8-min session. After every mouse was tested, the maze was cleaned with 70% ethanol, removing any olfactory cues that may introduce errors into the observations. An actual alternation was defined as successive entries into all three arms, known as overlapping triplet sets. Possible alternations were defined as the total number of arm entries. The percentage of spontaneous alternation behavior was calculated as the ratio of actual alternations to possible alternations multiplied by 100.

#### Elevated Plus Maze Test (EPMT)

The elevated plus maze test is used to assess anxiety-related behavior in rodent models of CNS disorders. The elevated plus maze is a plus shaped apparatus with four arms at right angles to each other as described by Handley and Mithani [35]. The two open arms lie across each other measuring 25 × 5 × 5 cm and perpendicular to two closed arms measuring 25 × 5 × 16 cm with a central platform (5 × 5 × 0.5 cm). The closed arms have a high wall (16 cm) to enclose the arms whereas the open arms have no side wall. The task is based on an approach-avoidance conflict, meaning that the animal is faced with a struggle between a propensity to explore a novel environment and an unconditioned fear of high and open spaces. Consequently, an anxiety-like state is characterized by increased open arm avoidance, compared to control animals. Rats were placed in the central platform facing the closed arm and their behavior recorded for 5 min. The criterion for arm visit was considered only when the animal decisively moved all its four limbs into an arm. The maze was cleaned with 70% ethanol after each trial. The elevated plus maze relies upon rodents’ proclivity toward dark, enclosed spaces (approach) and an unconditioned fear of heights/open spaces (avoidance) [36]. The percentage of time spent in the arms was calculated as time in open arms or closed arm/total time × 100, the number of entries into the arms was calculated using number of entries into open or closed arms/total number of entries.

#### Open Field Test (OFT)

The open field apparatus was constructed of white plywood and measured 72 × 72 cm with 36 cm walls. One of the walls was clear Plexiglas, so rats could be visible in the apparatus. Blue lines were drawn on the floor with a marker and were visible through the clear plexiglas floor. The lines divided the floor into sixteen 18 × 18 cm squares. A central square (18 cm × 18 cm) was drawn in the middle of the open field. Rats were placed individually in the center of the open-field and behavioral parameters were assessed manually for 10 min. Four motor parameters were quantified throughout this test: central square duration (the duration of time the rats spent in the central square), line crossings (the number of times the rats crossed one of the grid lines with all four paws), rearing (the number of times the rats stood on their hind legs in the maze) and freezing time (duration in which the rat was completely stationary). The most established indicators of emotional behavior in the open field test are ambulation and defecation [37]. It has been proposed that fear response (or anxiety) of the animal exposed to a new and thus potentially dangerous environment is accompanied by high defecation as well as by low ambulation, especially in the central zone [38]. The open field apparatus was cleaned after each session using 70% ethanol and permitted to dry between tests [39].

Hall’s experiment for determining the relationship between emotional behavior and the speed of ambulatory activity in an open-field task suggested that emotional rats tended to be less active than their non-emotional counterparts [40].

### Neurochemical Analyses

#### Determination of lipid Peroxidation Level

The lipid peroxidation marker (MDA) was evaluated in the cortical homogenates of different groups by measuring malondialdehyde level that represents one of the marked metabolites of lipid peroxide. The principal of this method is based on the procedure described by Ruiz-Larrea et al. [41]. It depends on the interaction between malondialdehyde and thiobarbituric acid in acidic medium placed in boiling water bath for 20 min. As a result of this interaction, a pink color is produced. The absorbance of the output derivative was read at 532 nm using UV–visible spectrophotometer.

#### Determination of Nitric Oxide Level

Nitric oxide (NO) level was evaluated in the cortical homogenates by measuring nitrite level. In this method, Griess reagent reacts with nitrite forming a deep purple colored compound. The absorbance of this compound indicates NO level and is read at 540 nm [42].

#### Determination of Reduced Glutathione Level

Reduced glutathione (GSH) was measured spectrophotometrically. The method is based on the reduction of 5,5′ dithiobis (2–nitrobenzoic acid) with glutathione (GSH) to produce a yellow compound. The reduced chromogen is directly proportional to GSH concentration and its absorbance is measured at 405 nm [43].

#### Determination of Monoamine Oxidase Level

The quantitative determination of rat monoamine oxidase (MAO) concentrations in tissue homogenates was carried out using rat monoamine oxidase ELISA Kit (SinoGeneclon Co., Ltd Catalog NoSG-20929). This assay employs the quantitative sandwich enzyme immunoassay technique. Antibody specific for MAO has been pre-coated onto a microplate. Standards and samples are pipetted into the wells and any MAO present is bound by the immobilized antibody. After removing any unbound substances, a biotin-conjugated antibody specific for MAO is added to the wells. After washing, avidin-conjugated horseradish peroxidase (HRP) is added to the wells. Following a wash to remove any unbound avidin-enzyme reagent, a substrate solution is added to the wells and color develops in proportion to the amount of MAO bound in the initial step. The color development is stopped and the intensity of the color is measured.

#### Determination of Acetylcholinesterase Level

Acetylcholinesterase (AChE) was assayed quantitatively using ELISA Kit (SinoGeneclon Co., Ltd Catalog NoSG-20512). This assay employs the quantitative sandwich enzyme immunoassay technique. The antibody specific for AChE has been pre-coated onto a microplate. Standards and samples are pipetted into the wells and any AChE present is bound by the immobilized antibody. After removing any unbound substances, a biotin-conjugated antibody specific for AChE is added to the wells. After washing, avidin-conjugated horseradish peroxidase (HRP) is added to the wells. Following a wash to remove any unbound avidin-enzyme reagent, a substrate solution is added to the wells and color develops in proportion to the amount of AChE bound in the initial step. The color development is stopped and the intensity of the color is measured.

#### Detection of Bcl2, Caspase-3and Inducible Nitric Oxide Synthase (iNOS) by Western Blot Technique

For Western blot analysis, cortical tissues were homogenized in buffer consisting of 50 mMTris pH 7.4, 10 mMNaF, 2 mM EDTA, 10 mM β-glycerol phosphate, 1 mM Na_3_VO_4_, 0.2% W/V sodium deoxycholate, 1 mM phenyl methyl sulfonyl fluoride (PMSF), and complete protease inhibitor cocktail (Sigma, P8340) employing polytron homogenizer (POLYTRON_ PT 10–35, Kinematica, Switzerland) in ice. The lysates were centrifuged at 4 °C for 15 min at 10,000 g. Protein concentration of the supernatants was determined using the Bradford protein assay kit (Bio-Rad). Levels of Bcl-2, caspase-3, iNOS and β-actin were measured by immune blotting analysis. Briefly, prepared samples were mixed with loading buffer and heated for 8 min at 95 °C, separated by sodium dodecyl sulfate polyacrylamide gel electrophoresis on a 12% gel, and then transferred to polyvinylidene fluoride (PVDF) membrane. The blots were incubated in blocking buffer TBS-T (5% nonfat milk and 0.1% Tween-20 in Tris-buffered saline) for 2 h at room temperature. The blots were developed using the primary antibodies against Bcl-2 (Cell Signaling, #2870), caspase3 (Cell Signaling, #9665), iNOS (Cell Signaling, #4790), and β-actin (Cell Signaling, # 3700). All antibodies were used at a dilution of 1:1000. The blots were incubated with the primary antibodies at 4 °C overnight and then washed three times for 10 min each in TSB with 0.1% Tween-20. Finally, the membranes were incubated with the corresponding secondary antibody and washed as described above. The protein bands were detected. The analysis was conducted by using software of Bio-Rad Laboratories Inc. (Hercules, CA, USA). All protein bands were normalized against β-actin protein [44].

### Histological Examination

Tissue samples were flushed and fixed in 10% neutral buffered formalin for 72 hs. Samples were processed in serial grades of alcohols, and cleared in xylene. Then, samples were infiltrated and embedded into paraplast tissue embedding media. 4μn thick sagittal brain sections were cut by rotatory microtome for demonstration of cortical regions in different groups. The sections were stained by hematoxylin and eosin for general morphological examination and stained by toluidine blue for demonstration of damaged and intact neurons then examined by using light microscope (Leica Microsystems GmbH, Wetzlar, Germany). All standard procedures for samples fixation and staining were carried out according to Culling [45]. Six non-overlapping fields of cerebral cortex (magnification × 400) were randomly selected and scanned for the determination of numbers of intact neurons in toluidine blue-stained tissue sections. Morphological examination and data analysis were carried out using Leica Application module for tissue sections analysis attached to Full HD microscopic imaging system (Leica Microsystems GmbH, Germany).

### Statistical Analysis

Data were analyzed statistically using one-way ANOVA followed by Duncan as post hoc test to compare between different groups. The difference between groups was considered significant at p-value < 0.05. Data are expressed as mean ± SEM.

## Results

### Behavioral Results

#### Spontaneous Alternation of Behavior in Y-Maze

**Fig. 1:**
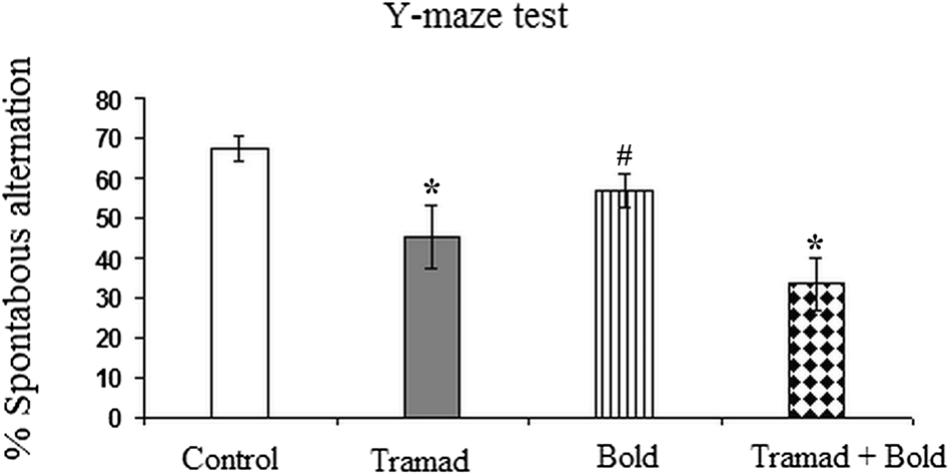
Effect of Boldenone and /or tramadol on the spontaneous alternation percent of Y-maze test in rats Control rats, Rats treated with Boldenone, Rats treated with tramadol, Rats cotreated with boldenone and tramadol . Data are expressed as mean ± SEM. The sign * indicates a significant change as compared to control value at p-value < 0.05. the sign # indicates significance changes in comparison to the other groups. Same letters indicate non-significant changes.

Rats treated daily with tramadol showed a significant decrease in percent of spontaneous alternation as compared to control animals (p-value 0.002, F = 7.434). However, rats treated with boldenone exhibited non-significant changes as compared to control rats and tramadol- treated rats. When the rats were treated with tramadol and boldenone, the percent of alternation decreased significantly as compared to control (p-value 0.002, F = 5.029) (Fig. 1).

#### Results of Elevated Plus Maze Test

**Fig. 2:**
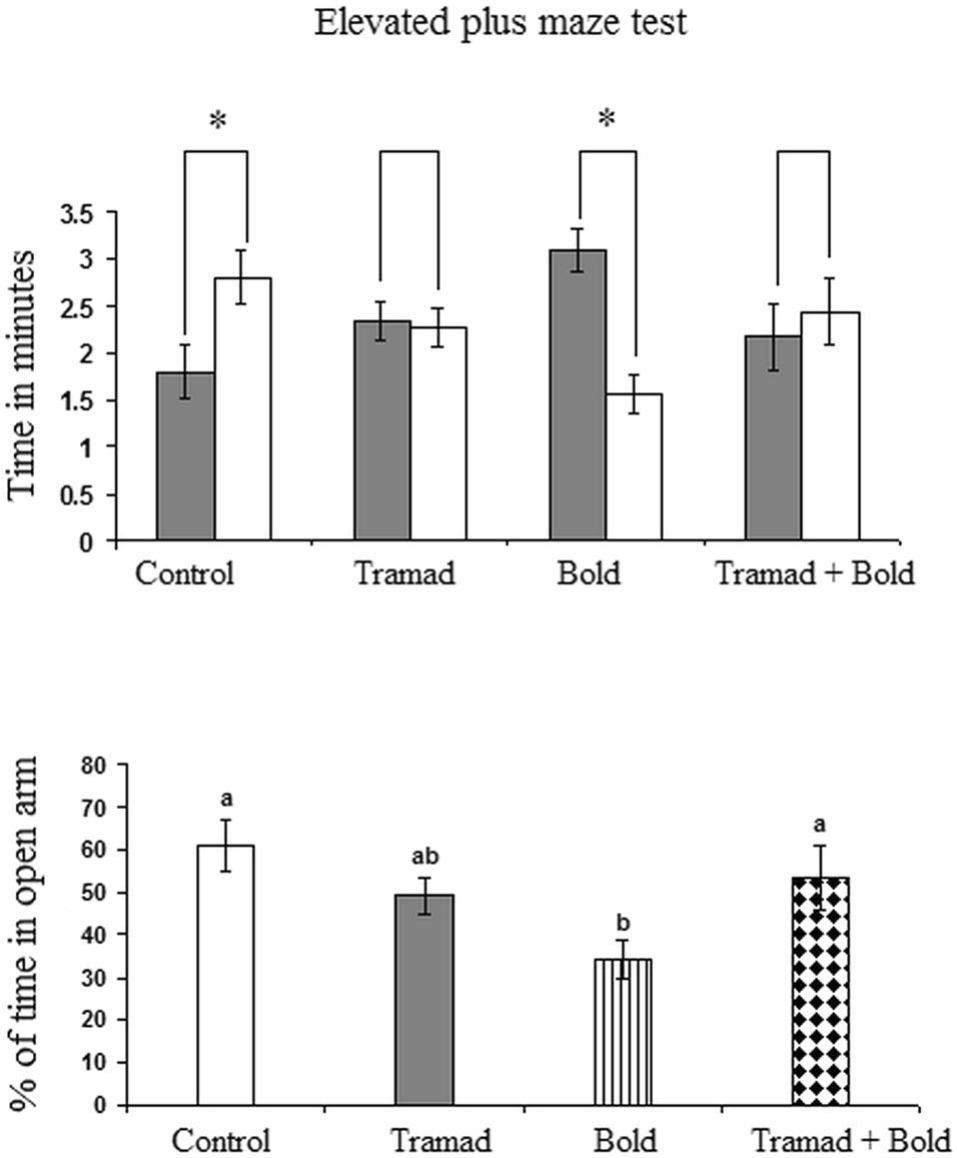
Effect of Boldenone and/or tramadol on the time in minutes spent by rats in the open arm and closed arm in elevated plus maze test Control rats, , Rats treated with Boldenone , Rats treated with tramadol , Rats cotreated with boldenone and tramadol . Data are expressed as mean ± SEM. Different letters indicate significantly different means at p-value < 0.05. Same letters indicate non-significant changes.

Control rats showed a significant increase in the time spent in the open arm as compared to the time spent in the closed arm (p-value 0.0.036, F = 6.329). However, the time spent in the open arm decreased significantly in rats treated with boldenone (p-value 0.012, F = 4.565). Rats treated with tramadol or both drugs did not record any change in the time spent in either closed or open arms (Fig. 2). The percent of time spent in the open arm after 5 min of exploration in elevated plus maze test showed a significant decrease in rats treated with boldenone (p-value 0.013, F = 4.478). However, no significant changes were recorded in rats treated with tramadol only or tramadol and boldenone (Fig. 2).

#### Open Field Data

**Fig. 3:**
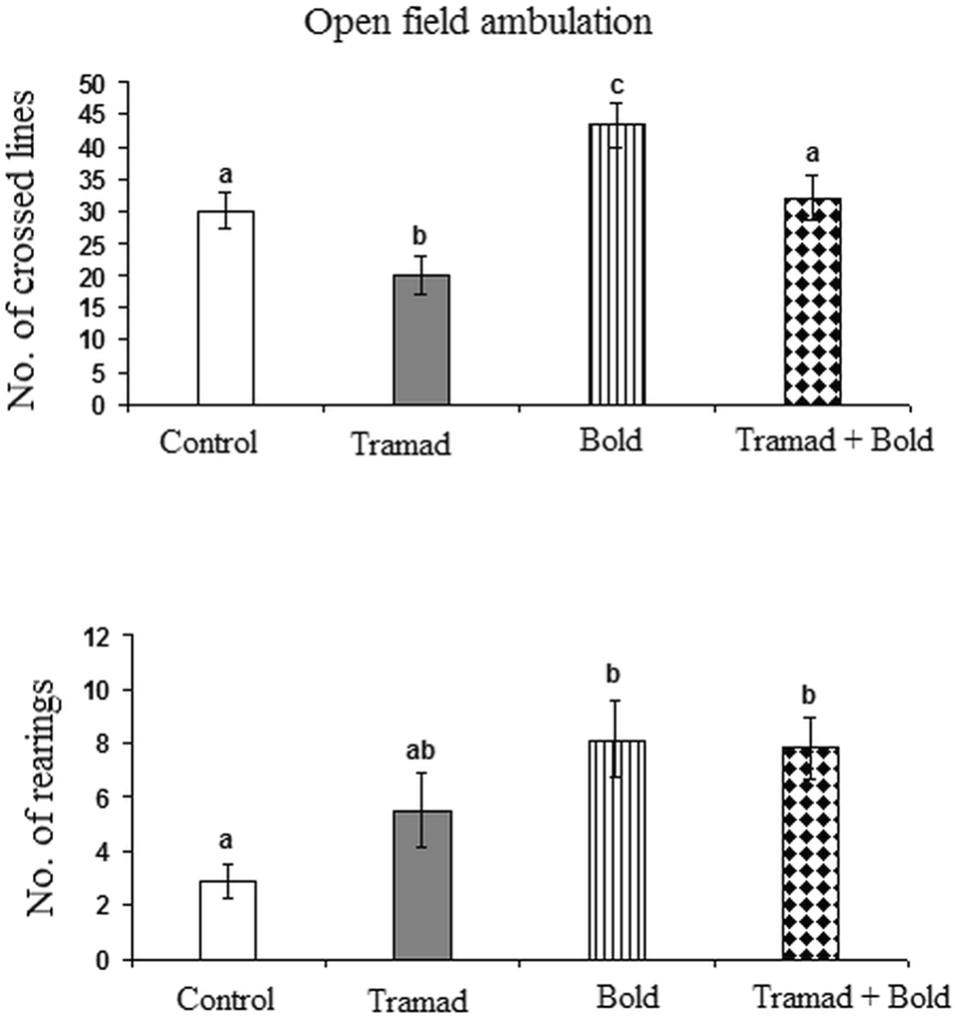
Effect of Boldenone and /or tramadol on the open field test parameters Control rats Rat treated with boldenone , Rat treated with tramadol , Rat co-treated with boldenone and tramadol . Different letters indicate significantly different means at p-value < 0.05. Same letters indicate non-significant changes.

As shown in Fig. 3, tramadol reduced ambulation significantly while boldenone treatment resulted in a significant increase (p-value 0.00, F = 11.579). Rats treated with tramadol and boldenone did not show any changes in ambulation. Number of rearings increased significantly in rats treated with boldenone alone or with tramadol as compared to control (p-value 0.008, F = 5.034). No significant changes were recorded in the number of rearings of rats treated with tramadol as compared to control and boldenone-treated rats.

### Neurochemical Data

#### Effect of Tramadol and/or Boldenone on Lipid Peroxidation, Nitric Oxide and Reduced Glutathione

**Fig. 4:**
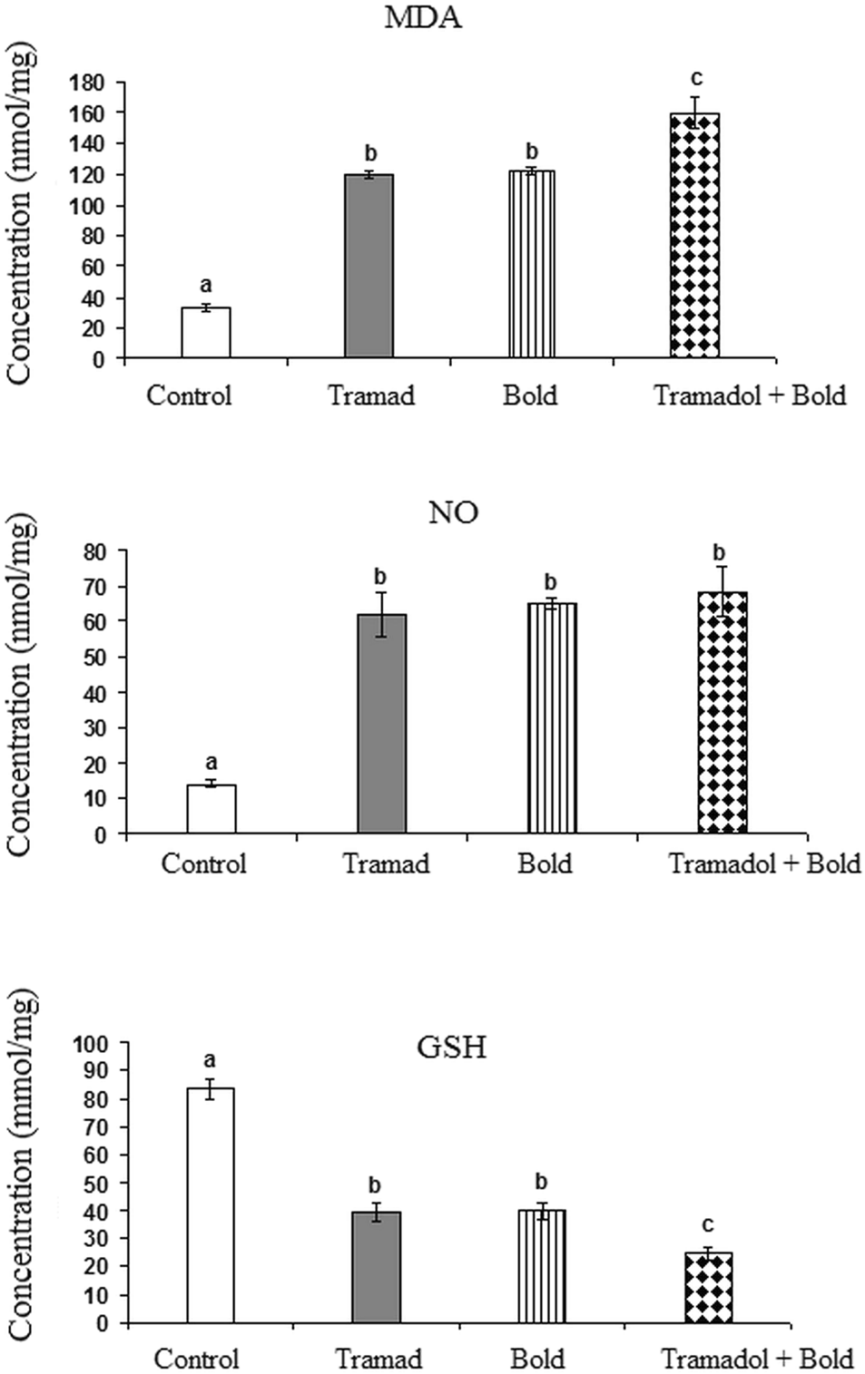
Effect of Boldenone and /or tramadol on the levels of lipid peroxidation (MDA), nitric oxide (NO) and reduced glutathione (GSH) in the cortex of rats Control rats , Rat treated with boldenone , Rat treated with tramadol , Rat co-treated with boldenone and tramadol . Different letters indicate significantly different means at p-value < 0.05. Same letters indicate non-significant changes.

Treatment of rats with boldenone and /or tramadol increased the level of MDA in the cortex significantly as compared to control (p-value 0.0001, F = 88.176). The greatest increase was recorded in rats treated with both drugs showing a significant increase as compared to control, tramadol-treated rats and boldenone-treated rats. This was associated with a significant increase in the level of NO in rats treated with tramadol and/or boldenone (p-value 0.0001, F = 28.793). However, cortical GSH decreased significantly in rats treated with tramadol alone or combined with boldenone as compared to control rats (p-value 0.0001, F = 61.231). The decreased GSH level recorded in rats treated with both drugs was significantly lower than control, tramadol-treated rats and boldenone-treated rats (Fig. 4).

#### Effect of Tramadol and/or Boldenone on the Levels of Bcl2, Caspase-3 and Inducible Nitric Oxide Synthase (iNOS)

The present data revealed that treatment of rats with boldenone and/or tramadol reduced the level of cortical Bcl2 significantly as compared to control value (p-value 0.0001, F = 95.870). In addition, the decrease in Bcl2 in rats treated with the two drugs together was significantly lower as compared to rats treated with each drug alone.

**Fig. 5:**
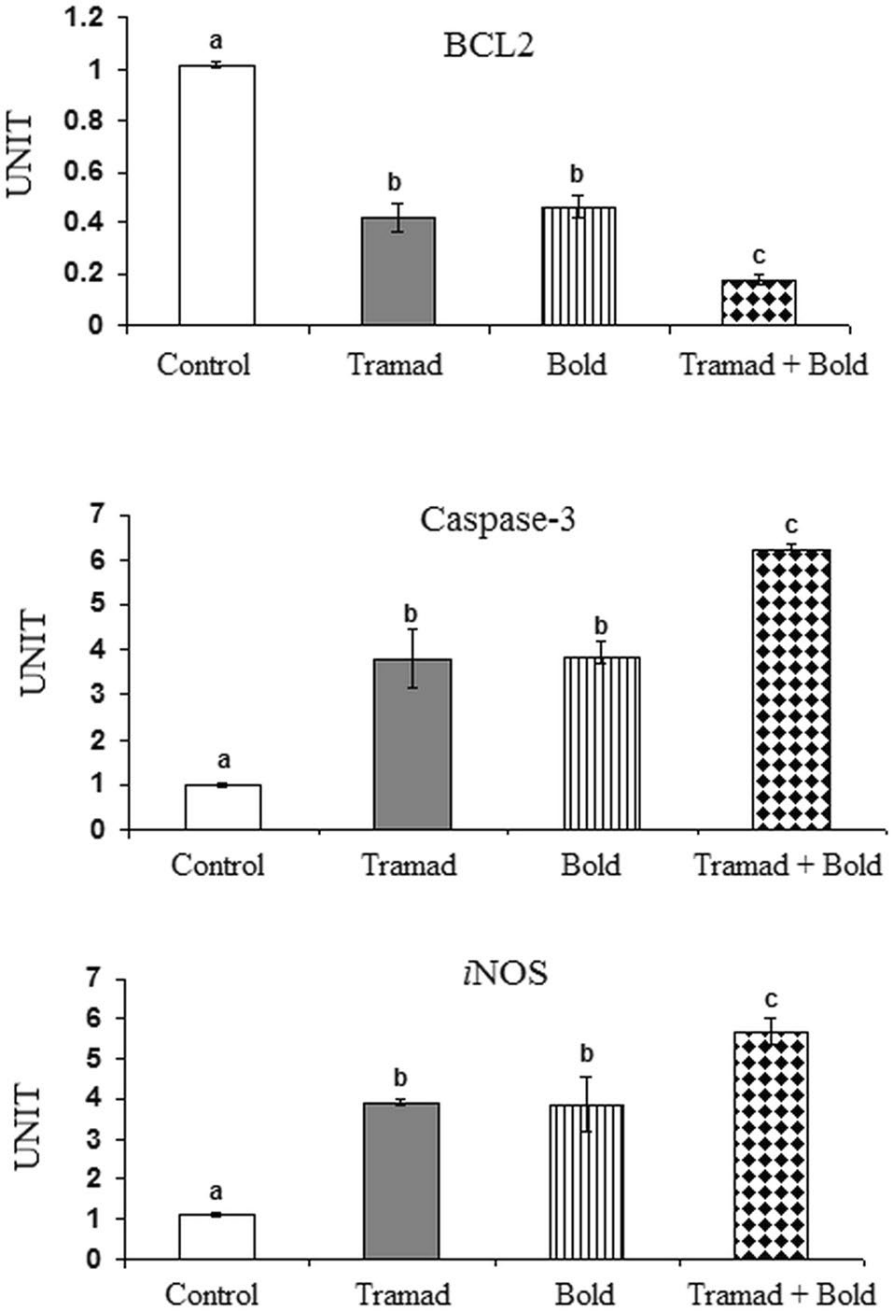
Effect of Boldenone and /or tramadol on the levels Bcl2, caspase-3 and inducible nitric oxide synthase (iNOS) and in the cortex of rats Control rats , Rat treated with boldenone , Rat treated with tramadol , Rat co-treated with boldenone and tramadol , Different letters indicate significantly different means at p-value < 0.05. Same letters indicate non-significant changes.

Caspase-3 and iNOS levels increased significantly in the cortex of rats treated with tramadol, boldenone, or both drugs as compared to control (p-value 0.001, F = 31.961 and p-value 0.001, F = 26.319, respectively). Their increased levels were more prominent in rats treated with the two drugs being significant as compared to tramadol-treated rats and boldenone-treated rats (Fig. 5).

#### Effect of Tramadol and/or Boldenone on the Levels of Acetylcholinesterase (AChE) and Monoamine Oxidase (MAO)

**Fig. 6:**
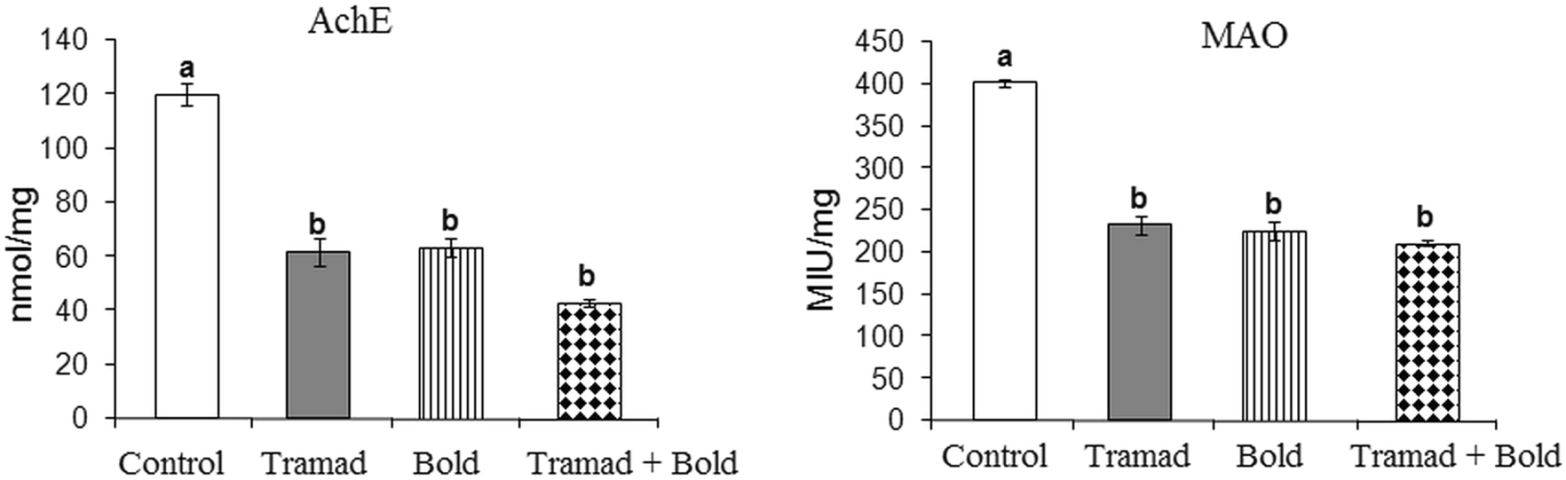
Effect of Boldenone and/or tramadol on the concentration of acetylcholinesterase (AChE) and monoamine oxidase (MAO) in the cortex of rats Control rats , Rats treated with bole none , Rats treated with tramadol , Rats co-treated with boldenone and tramadol . Different letters indicate significantly different means at p-value < 0.05. Same letters indicate non-significant changes.

Animals treated with boldenone and/or tramadol showed a significant decrease in the levels of AChE and MAO as compared to control values (p-value 0.0001, F = 7.730 and p-value 0.001, F = 15.151, respectively) (Fig. 6).

### Histopathological Changes Induced by Tramadol and/or Boldenone

**Fig. 7:**
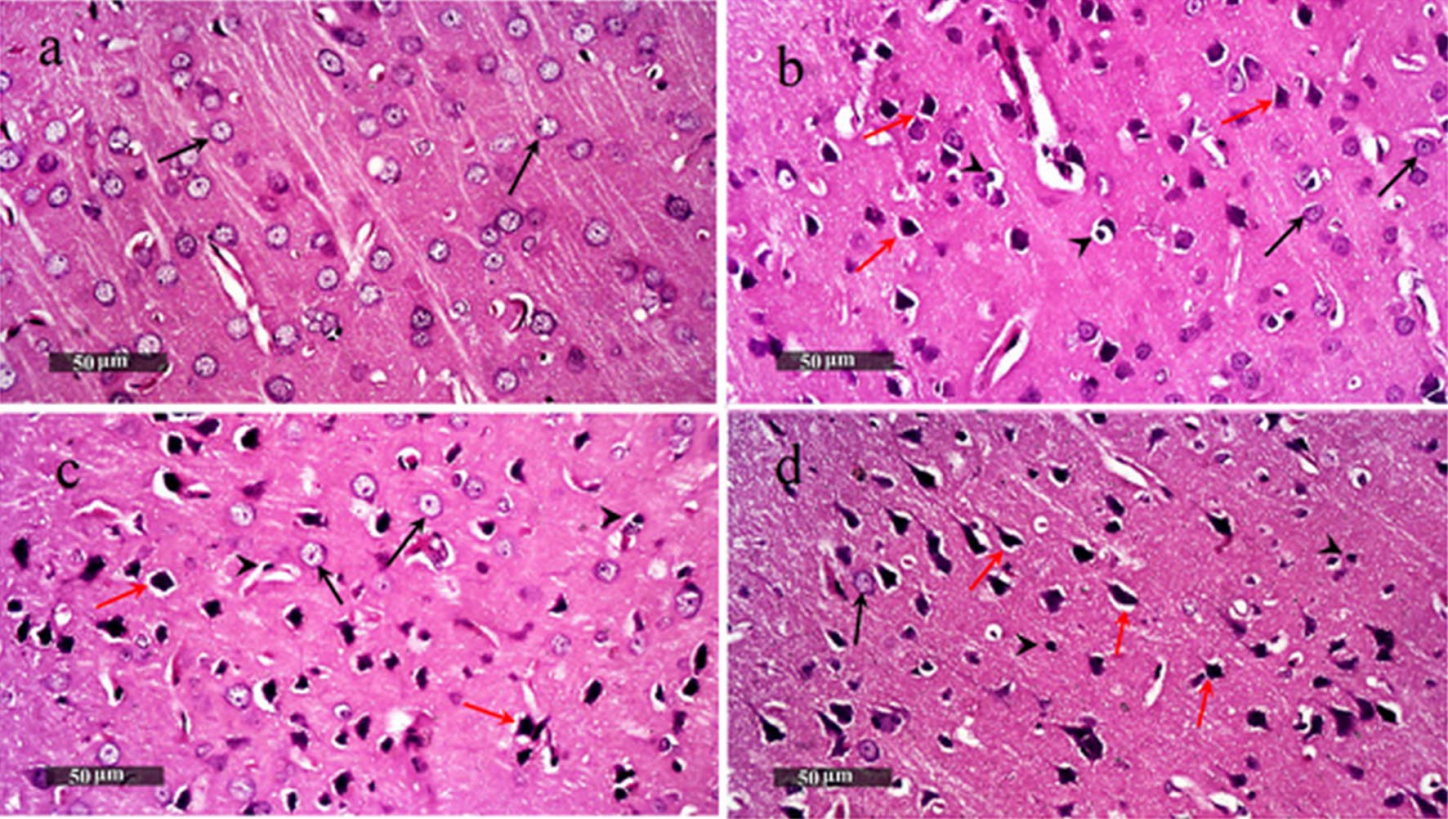
Histological changes induced by Boldenone and /or tramadol in the cortex sections stained by hematoxylin and eosin (H&E × 400). The cerebral cortex of control rats demonstrates normal histological features with many well-organized apparent neurons having intact cell membranes (arrows). Minimal glial cells with small dense nuclei (**a**). Focal areas with neuronal degenerative changes (red arrow) alternating with apparent intact neurons (black arrow) and moderate accumulation of glial cells with small dense nuclei (arrow head) and minimal congested blood vessels were observed in the cerebral cortex of rats treated with either boldenone (**b**) or tramadol (**c**). In rats co-treated with boldenone and tramadol, more diffuse patterns of neuronal damage and degenerative changes (red arrow) with fewer apparent intact neurons were shown (black arrow) accompanied by numerous glial cells with small dense nuclei (arrow head) (**d**) (Color figure online)

**Fig. 8:**
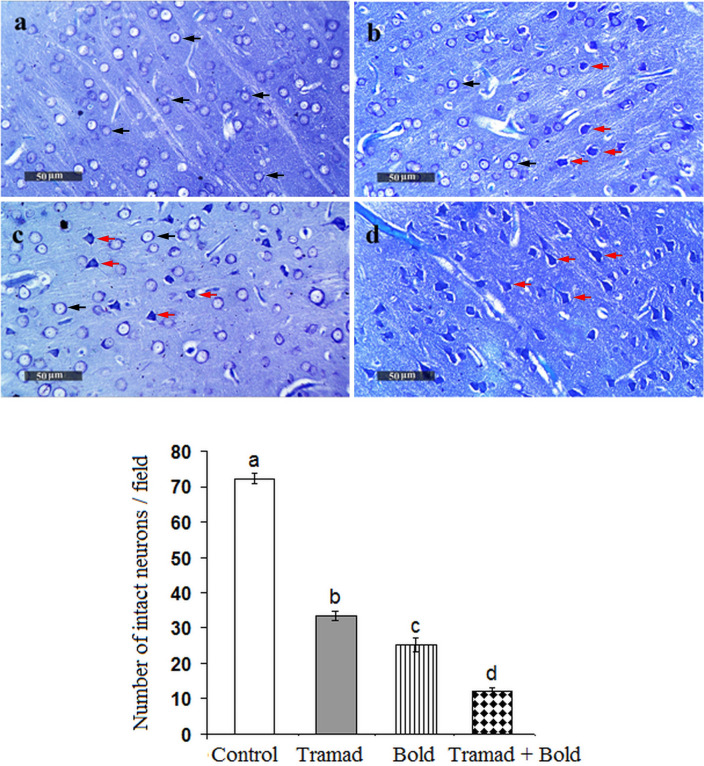
Sections of the cerebral cortex stained by toluidine blue showing number of the intact (black arrow) and damaged neurons (red arrow) induced by boldenone and/or tramadol. Histological examination of the cortex shows a decreased number of intact neurons in rats treated with tramadol (Fig. 8b), boldenone (Fig. 8c) or both agents (Fig. 8d) as compared to control (Fig. 8a) (Color figure online)

Microscopic examination of the cerebral cortex of control rats showed normal histological features of cerebral cortical layers with many well organized apparent intact neurons having intact cell membrane. The glial cells are intact and there is no sign of cytoplasmic shrinkage (arrows) (Fig. 7a). The cerebral cortex of rats treated with tramadol exhibited focal areas with neuronal degenerative changes (red arrow) alternating with apparent intact neurons (black arrow). Moderate accumulation of glial cells was observed (arrow head) with minimal congested blood vessels (Fig. 7b). The cerebral cortex of rats treated with boldenone showed similar histological changes (Fig. 7c). The cerebral cortex of rats treated with tramadol and boldenone showed more diffuse patterns of neuronal damage and degenerative changes (red arrow) with fewer apparent intact neurons (black arrow) accompanied by numerous glial cells with small dense nuclei (arrow head) (Fig. 7d). Examination of the cerebral tissue stained with toluidine blue showed that the number of intact neurons decreased significantly in rats treated with tramadol (Fig. 8b), boldenone (Fig. 8c) or both agents (Fig. 8d) as compared to control rats (Fig. 8a). The number of intact neurons in the cerebral cortex of rats co-treated with tramadol and boldenone was lower than those in rats treated with either tramadol or boldenone (Fig. 8).

## Discussion

Due to the increased numbers of boldenone and tramadol abusers, the present study was conducted to evaluate their impacts either solely or in combination on memory, cognition and emotional state of rats. In addition, the neurochemical changes induced by these agents were assessed.

The present study revealed that rats treated with boldenone and/or tramadol showed a decline in short-term memory. This was indicated from the significant decrease in the percentage of spontaneous alternation in YMT. This test evaluates both the exploratory and cognitive behaviors associated with spatial learning and memory using the spontaneous alternation behavior as an indicator of spatial working memory, a form of short-term memory [46].YMT depends on native tendency of rats to explore the new arm of the maze rather than return to the same arm they previously visited [47]. The cerebral cortex is one of the brain regions contributing potentially in this task [48] that can assess cognitive deficits and drug effects on cognition [49]. In addition, the present elevated plus maze test (EPMT) analyses showed a decrease in the time spent by rats treated with boldenone in the open arm. It has been reported that anxiety-like behavior may be manifested by the immobility of the animal positioned in the elevated plus maze and its avoidance of the open arm [50]. EPMT is used widely to examine anxiety-like behavior [31] which is determined by the ratio of the time spent in the open arms to the time spent in the closed arms [50]. The present findings indicate that boldenone could potentially precipitate anxiety more than tramadol. Surprisingly, when the two agents, tramadol and boldenone, were administered together, the level of anxiety in rodents returned to control values as indicated from the increase in the time spent in the open arms. This effect could be attributed to the anxiolytic activity of tramadol mediated by its effect on monoamine and opioid levels [51]. Therefore, the anxiety induced by boldenone may be reversed by tramadol. Tramadol has various mechanisms of action. It causes sedation by the inhibition of serotonin and noradrenaline reuptake transporters in the CNS [52] as antidepressant do. In rodent models, tramadol decreased anxiety and thus the animals investigated their open environment more in the elevated plus maze test [53]. Moreover, the study of Rojas-Corrales et al. suggested that the antidepressant-like effect of tramadol in mice was mediated by the noradrenergic system, rather than the serotonergic or opioidergic system [54]. Numerous studies have showed that the reduced monoamine oxidase activity predisposed anxiety and aggressive behavior [55, 56] and serotonin syndrome which could be attributed to the increase in serotonin level [57]. Therefore, the cortical decrease in MAO activity induced by boldenone and /or tramadol could mediate the reported aggressive behavior and serotonin syndrome exerted by the two agents. These effects may make their abusers more susceptible to anger by reducing the anxiety threshold [13].

The present open field data indicate that boldenone increased motor activity. This was elucidated from the increased ambulation and number of rearings. This effect was expected due to the effect of boldenone on muscle performance. These results may also be attributed to an increase in the aggressiveness and activities under the effect of boldenone [13]. Aggression, anxiety, and reproductive behaviors involving GABAergic transmission are mainly affected by AAS use in both human abusers and animal models [58]. However, tramadol reduced ambulation. The present data showed that the increased ambulation induced by boldenone reversed the decreased ambulation induced by tramadol when the two drugs were co-administered together leading to a normal-like effect. Rearing might be a more direct measure of anxiety [59]. It consists of animals standing on both hind paws in a vertical upright position and is considered an exploratory behavior [60, 61]. Therefore, the increased rearing number induced by boldenone supports its anxiogenic-like effect. This effect was absent in case of tramadol due to its antidepressant and calming effects. However, when the two drugs were co-administered, tramadol couldn’t counteract the increase in the number of rearings induced by boldenone.

The present increase in the levels of lipid peroxidation and nitric oxide together with the reduced GSH level indicate that boldenone and/or tramadol induced a state of oxidative stress in the cerebral tissue which is highly sensitive to oxidative stress due to its high metabolic rate, its enrichment in lipids and low content of antioxidants [62]. This effect could contribute to the neurodegeneration induced by these agents. Lipid peroxidation is one of the major effects of free radical-induced damage that directly targets the neuronal membranes yielding several secondary products that extend the cellular injury [63]. Therefore, lipid peroxidation is used as a marker of free radical-mediated oxidative damage [64] that has been implicated in several neurodegenerative diseases. In addition to their damaging effect on membrane phospholipids, free radicals can directly attack membrane proteins forming lipid-protein and protein–protein cross links, all of which contribute to altered membrane integrity [65]. This action deteriorates the neuronal homeostasis and contributes to the brain dysfunction [63].

Nitric oxide is one of the free radicals that can combine with superoxides forming peroxynitrite which has a potent damaging effect in the CNS [66]. Nitric oxide synthase (NOS) is the enzyme responsible for synthesizing NO from L-arginine [67]. In the CNS, three NOS isoforms have been recognized; inducible NOS (iNOS), endothelial NOS (eNOS) and neuronal NOS (nNOS) [68] which have different patterns of activity. iNOS is a calcium-independent isoform often produced by microglia and astrocytes in pathological conditions, nNOS is distributed in synaptic spines, astrocytes and the loose connective tissue surrounding the brain blood vessels [69] and eNOS is located in the cerebral vascular endothelial cells and motor neurons [70]. The activity of iNOS is induced upon stimulation by proinflammatory cytokines over a long period of time [71]. Simultaneous production of NO and superoxide by activated microglia, under proinflammatory conditions, gives rise to the formation of peroxynitrite that induces neuronal death [72]. Thus, the present increase in iNOS in the cortex under the effect of boldenone and tramadol could be responsible for the significant increase in cortical NO. This may represent one of the mechanisms that underlie the neuroinflammation and neurodegeneration induced by tramadol and boldenone.

Reduced glutathione is the most important free radical scavenger in the cortex [73]. Thus, its reduced level after the administration of boldenone and /or tramadol could be due to its consumption in scavenging the produced free radicals. The reduced level of GSH has been recorded in many neurodegenerative diseases. Therefore, the present deficiency in GSH content together with the elevated levels of NO and MDA may indicate the induction of neurodegeneration by boldenone and/or tramadol. Supporting this notion is the reduced level of Bcl2 and the elevated level of caspase-3 that have been recorded in the cortex of rats treated with either of these two agents or their combination.

Bcl-2 can protect cells from various insults. BCL-2 overexpression protected cortical neurons and reduced the hippocampal lesion size mediated by NMDA overstimulation and the subsequent neurotoxic damage [72]. Bcl-2 deficient mice showed increased oxidative stress and changes in brain antioxidant mechanism [73] while Bcl-2 up-regulation could help in DNA repair after oxidative stress and maintain cell survival [74].

Caspase -3 is the key enzyme in apoptosis implementation [75] which is normally present in a quiescent state. It is activated by inflammatory factors released by brain tissue during cerebral ischemia or cerebral ischemia–reperfusion [76] leading to apoptosis. The present elevated caspase 3 may be induced as a consequence of the oxidative stress induced by these agents. Coimbra-Costa et al. suggested that the cerebral activation of caspase 3 could be a delayed effect of oxidative stress in the induction of apoptosis [77]. In addition, free radicals increase the permeability of the mitochondrial membrane by rupturing the outer membrane of the mitochondria releasing substances such as apoptosis-inducing factor and cytochrome c that produce apoptotic cell death [78, 79].

It has been reported that down regulation of caspase-3 expression and up regulation of Bcl-2 expression can both inhibit neuronal apoptosis produced by cerebral ischemia [80]. Therefore, the reduced Bcl-2 and the increased caspase-3 levels induced by boldenone and/or tramadol treatment in the present study could promote cortical apoptosis and cell loss and this may explain the impairment in memory and cognition induced by boldenone and/or tramadol.

AChE is an important enzyme that regulates the concentration of acetylcholine in the synaptic cleft. Cholinergic activity has a crucial role in memory and cognitive functions [81].On the other hand, the decreased activity of AChE may account for cholinergic hyperactivity which may results in status epilepticus and convulsions [82].Supporting this assumption is the decrease in cholinergic activity as indicated from the present reduction in cortical AChE level which is correlated with the level of its substrate, acetylcholine. Loss of brain AChE activity is evident in several brain disorders including neurodegenerative diseases as AD [83] where the decrease of AChE in the cortex and hippocampus has been extensively reported [83, 84]. Reduced AChE activity together with mitochondrial dysfunction, and inflammation are the prime causes of impaired memory and cognitive impairment [85]. Reduction in AChE activity, a marker of cholinergic activity in cognitive and memory impairment disorders has been established [86]. In addition, positron emission tomography and autopsy studies revealed a loss of AChE in the forebrain of Alzheimer’s disease patients [87, 88]. The present decreased AChE activity could be ascribed to the oxidative stress observed in the cortex. AChE activity is sensitive to free radicals and increased oxidative stress [89]. The decrease in AChE has also been observed with the increase in lipid peroxidation during human aging [90].

Histological examination showed a decrease in the number of survived neurons in the cerebral cortex of rats treated with tramadol and/or boldenone. The animals treated with both agents were drastically affected. The neuronal cell death induced by tramadol and/or boldenone in the cortex of rats could be attributed to the free radical production that can attack the DNA, protein and phospholipids in the cell membrane. Nitric oxide could have a pivotal role in the damaging effect through the formation of peroxynitrite, the powerful oxidant and damaging agent. Moreover, the elevated level of caspase-3 acts as a key executioner of apoptosis, being responsible for the proteolytic cleavage of many structural and regulatory proteins such as cytoskeletal proteins, nuclear proteins, and proteins involved in DNA metabolism [91]. Therefore, the histopathological changes induced in the cortical tissue could be mediated by the increase in free radical production and caspase-3 and decrease in Bcl-2 levels induced by tramadol and/or boldenone.

## Conclusion

According to the present data tramadol and /or boldenone induced neurotoxic effects mediated by increased oxidative and nitrosative stress, neuroinflammation and neuronal death in the cortex of rats. These effects resulted in impairment in memory and cognition. The current study is the first that investigates the adverse effects of the combined administration of tramadol and/or boldenone whose abuse prevails among adolescents. Although boldenone may be used among adolescents to build their muscles and obtain certain features, it may adversely affect their brains especially when boldenone is co-administered with tramadol.

The present findings warn against the abuse of boldenone and tramadol especially among athletes and adolescents who may have to use tramadol to relief the pain and the aggressiveness induced by boldenone.
